# Base excess (BE): reloaded

**DOI:** 10.1186/s40001-024-01796-6

**Published:** 2024-05-12

**Authors:** Rolf Zander

**Affiliations:** Physioklin, formerly Institute of Physiology and Pathophysiology, Mainz University, Mainz, Germany

**Keywords:** Base excess, Intensive care diagnostic, Mortality, Shock, Acidosis, Bleeding, Artificial ventilation, Infusion solutions, Potential base excess, Blood products

## Abstract

The base excess value (BE, mmol/L), not standard base excess (SBE), correctly calculated including pH, pCO_2_ (mmHg), sO_2_ (%) and cHb (g/dl) is a *diagnosti*c tool for several in vivo events, e.g., mortality after multiple trauma or shock, acidosis, bleeding, clotting, artificial ventilation. In everyday clinical practice a few microlitres of blood (arterial, mixed venous or venous) are sufficient for optimal diagnostics of any metabolic acidosis or alkalosis.

The same applies to a *therapeutic* tool—then referred to as potential base excess (BEpot)—for several in vitro assessments, e.g., solutions for infusion, sodium bicarbonate, blood products, packed red blood cells, plasma. Thus, BE or BEpot has been a parameter with exceptional clinical significance since 2007.

## Introduction

BE (mmol/l) is calculated routinely by each blood gas analyzer and is defined as the amount of strong acid that must be added theoretically to each liter of fully oxygenated blood to return the pH to 7.40 at a temperature of 37°C and a pCO_2_ of 40 mmHg. In everyday clinical practice a few microlitres of each type of blood sample, i.e., arterial, mixed venous or venous, are sufficient for optimal diagnostics. Any respiratory (pCO_2_) or metabolic (BE) acidosis or alkalosis can be diagnosed in this way. The base excess (BE) or base deficit (BD, negative BE) of arterial blood has been shown to be the best quantitative indicator of acute blood loss in animal models, outperforming 27 other hemodynamic parameters and laboratory chemistries (Waisman et al. 1993, cited in 18). Since 1990, four clinical trials enrolling about 8,000 patients with multiple injuries have demonstrated that BE on admission, compared with a large number of other parameters, is indeed the best prognostic indicator for mortality, complication rate, transfusions needs, etc. It has also been shown that a potential decrease in BE from hospital to ICU admission is a valid estimate of subsequent risk. Therefore, this outstanding value Base Excess (BE) was named a parameter with exceptional clinical significance as early as 2007 and must be taken into account in the daily clinical routine.

## Base excess—theory

Berend’s 2018 review article “Diagnostic use of base excess in acid–base disorders” [1] started another worldwide discussion about the item Base Excess (BE). However, since 2018, there have been several new publications, e.g., Langer et al. in 2022 [2]. These contain some errors as well as several incomplete sentences concerning base excess (BE) in acid–base disorders, which is what will be discussed in the following.

*The physiological approach, based on the renal and lung acid–base interaction* [1], is patently incomplete, because an overview with the title “The liver: The forgotten organ in acid–base balance” was published as early as in 1995 [3].

The physiological facts concerning elimination of acids as H^+^-ions are:

For lungs: 10 mmol per minute of CO_2_ (≈ H_2_CO_3_); for liver: 40 mmol per hour for lactic acid alone; for kidneys: 40–80 mmol per day in form of H_2_PO_4_^−^ as well as NH_4_^+^.

*“Standard base excess is one of the most extensively studied prognostic markers (…) and is provided worldwide by most commercial blood gas analyzers”* [1] may be one side of the medal. It is inarguable that *“standard BE is used widely in clinical studies and in clinical practice throughout the world.”*

It is correct that “*the nomenclature for base excess can be confusing”*; the explanation for this is given by several manufacturers:

In **2004**, manufacturers of blood gas analyzers (Bayer Vital, Fresenius MC, Instrumentation Laboratory, Nova Biomedical, Radiometer Germany and Roche Diagnostics) as well as experts in the field (e.g., Mertzlufft, Schaffartzik, Zander) met in Mainz (Germany) to define standard symbols and definitions related to base excess. All the attendees except Radiometer agreed that oxygen saturation (sO_2_) should be included in the calculation of Base Excess [4].

In **2019**, however, Radiometer Copenhagen issued an official statement—in response to a request from Zander—clarifying its position:

“Radiometer recognizes that clinicians may have different preferences when it comes to calculation of base excess and is now providing a number of different options in the Instruction for Use. The algorithms are:

- cBase(B) or ABE = Actual base excess in whole blood
- cBase(B, ox) = cBase(B) of fully oxygenated blood
- cBase(Ecf) or SBE = Recognized as the in vivo expression of base excess. It refers to a model of the extracellular fluid (one part of blood is diluted by two parts of its own plasma) and is calculated using a standard value for the hemoglobin concentration of the total extracellular fluid.
- Base(Ecf, ox) = cBase(Ecf) of fully oxygenated blood.”
  1. Blood Gas and pH Analysis and Related Measurements; Approved Guideline—Second Edition. CLSI document C46-A2. Wayne, PA: Clinical and Laboratory Standards Institute 2009
  2. Siggaard-Andersen O. The acid–base status of the blood. 4th revised ed. Copenhagen: Munksgaard, 1976.
  3. Siggaard-Andersen O, Wimberley PD, Fogh-Andersen N, Gøthgen ICH: Measured and derived quantities with modern pH and blood gas equipment: calculation algorithms with 54 equations. Scand J Clin Lab Invest 1988; 48, Suppl 189: 7–15
  4. Burnett RW, Noonan DC. Calculations and correction factors used in determination of blood pH and blood gases. Clin Chem 1974; 20: 1499—1506.
  5. Kofstad J: All about base excess—to BE or not to BE (2003) Radiometer Medical ApS: G. Wennecke—July 24th, 2019.

Comment on Radiometer’s Statement:

Obviously, Radiometer “provides a number of different options”, “because of clinicians may have different preferences”. Indeed, the anesthesiologist in the ICU at night, the clinician, is interested very much in finding the optimal BE value?

The origin of Radiometer’s special policy regarding the calculation of Base Excess, which neglects oxygen saturation (sO_2_), can be found in a 2001 quote by J. Kofstad: “*In the clinical situation, the influence of changes in sO*_*2*_* on calculation of cBase is of little importance and can be neglected”* [5].

Also in **2019**, Roche Diagnostics International Ltd issued an official statement—in response to a request by Zander—clarifying their position:

“In our current blood gas analysis systems [6], the Zander formula for Base Excess (BE_act_) is easily configured. BE_act_ defines base excess in blood at current oxygen saturation. In parallel to BE_act_, our systems also provide guideline-recommended calculations [1] for base excess in blood (BE) and for base excess in extracellular fluids (BE_ecf_). We recommend that the system software be configured according to the hospital's needs.

We are fully committed to consider new clinical guidelines and updated clinical practices in the development of new products. Due to regulatory constraints, we cannot currently prioritize BE_act_ over BE and BE_ecf_ in our current systems. We will consider BE_act_ as the prioritized formula for base excess determination in future developments, given that a careful evaluation shows the superiority of BE_act_ in clinical practice.

1. cobas b 123 POC system, cobas b 221 system, cobas b 121 system
2. Clinical and Laboratory Standards Institute CLSI C46-A2 Blood gas und pH analysis and related measurements; Approved guideline, second edition.”

Roche Diagnostics International Ltd

R. Jäggi, R. Reynolds—September 12, 2019

Comment on Roche’s statement:

Roche is waiting for a careful evaluation demonstrating the superiority of BE_act_ in clinical practice, i.e., BE correctly calculated including sO_2_ (%) of the blood sample.

## Arguments for a calculation of base excess (BE) including oxygen saturation

Authors [7] claim that, due to the lower pH and higher pCO_2_, base excess (BE) is always 1.5–2 mmol/L higher in venous blood (i.e., before lungs) than in arterial blood (i.e., after lungs). However, the BE reflects the metabolic, non-respiratory aspect of acid–base homeostasis, and there should be no significant difference in BE between venous and arterial blood samples, i.e., in the case of metabolically healthy lungs. The very unrealistic consequence of this would be that the lungs would have to generate 1.5–2 mmol/L H^+^ every minute (10,800–14,400 mmol H^+^ per day) to decrease the positive BE.

Rather, the reported difference in BE between venous and arterial blood is a method-related error resulting from use of non-optimal equations to calculate the BE. In fact, when using the modified Van Slyke equation as per Zander (13—Physioklin 1/31/2012):

$$ {\text{BE }} = \, \left( {{1}-0.0{143 }\cdot {\text{ cHb}}} \right) \, \cdot \, [\left\{ {0.0{3}0{4 }\cdot {\text{ pCO}}_{2} \cdot { 1}0^{{\text{pH }}-{6}.{1}} -{24}.{26}} \right\} \, + \, \left( {{9}.{5} + {1}.{63 }\cdot {\text{ cHb}}} \right) \cdot \, \left( {{\text{pH }}--{7}.{4}} \right)] \, - \, 0.{2 }\cdot {\text{ cHb }}\cdot \, \left( {{1}-{\text{sO}}_{2} } \right) $$

where cHb (the content of hemoglobin) is measured in g/100 mL and pCO_2_ in mmHg, the last term is a correction for oxygen saturation (sO_2_) as a fraction. This is crucial due to the fact that oxygenated Hb is a stronger acid than deoxygenated Hb (the basis of the famous Christiansen-Douglas-Haldane effect, i.e., oxygenation of the blood expels the CO_2_ from blood into the alveoli).

If one equilibrates a blood sample with a cHb of 15 g/dL at pCO_2_ 40 mmHg and sO_2_ 100% down to a sO_2_ of 0%, then the pH rises from 7.400 to 7.441, and the remaining BE is constant at 0 mmol/L.

Therefore, the statement “*Measurement of standard base excess usually requires arterial blood, which may be difficult to obtain in the acute care setting. Venous blood can be obtained more easily and more rapidly, and values for venous standard base excess generally correlate well with arterial values”* [1] must be rejected.

Objection: Using the Zander Eq. (5), BE can be obtained with very high accuracy from any blood sample, venous or arterial, and, over a wide range of BE (-30 to + 30 mmol/L), the mean inaccuracy is less than 1 mmol/l.

The corresponding proof has been published [8]: typical measured results (mean ± SD) as obtained from blood from a cubital vein (50 healthy volunteers: colleagues and medical students) are pH 7.352 ± 0.023, pCO_2_ 51.2 ± 4.9 mmHg, pO_2_ 28.6 ± 10.2 mmHg, sO_2_ 49.2 ± 22.0%, and calculated BE as a mean was –0.1 ± 1 mmol/L. This shows that the venous and arterial base excess difference is a methodological error only [9].

Increasing base deficit, i.e., negative base excess, from venous to arterial:

As an aside, the only circulation (animal study) where the negative base excess increases from venous to arterial blood is hepatic portal circulation. pH increases by 0.02, pCO_2_ falls by 2 mmHg, lactate concentration is reduced by 0.44 mmol/L, and negative BE is decreased by 0.43 mmol/l [10]: the metabolism of the liver consumes CO_2_ and eliminates H^+^ for the oxidation of lactate, gluconeogenesis and synthesis of urea.

## Special statement on cotroverse O_2_ saturation

This is not a controversy but a statement of right or wrong or science versus business.

The story is easy to explain: One company among several others gives a statement and science contradicts it.

In 2001 quote by J. Kofstad, employees of Radiometer, one of the world market leaders:

“In the clinical situation, the influence of changes in sO_2_ on calculation of cBase [BE] is of little importance and can be neglected” [5].

In 2002 one year later, the answer from scientists [11]: Therefore, the equation according to Zander should be prefered to that originally proposed by Siggaard-Andersen, or by the National Committee for Clinical Laboratory Standards (NCCLS), and can be recommended.

for calculation of the whole blood base excess (BE) in the following form …

The advantage: The metabolic value BE can be obtained from any blood sample, arterial, mixed venous or venous in everyday clinical practice.

The disadvantage: If the sO_2_ is not taken into account, the BE before the lungs differs significantly from that after the lungs, which should not be the case for a metabolic value.

The result: The BE accordding to Gattinoni et al. [7] is always higher, by 1.5—2 mmol/l, in venous blood (i.e., before the lung) than in arterial blood (i.e., after the lung), owing to lower pH and higher pCO_2_ values [9].

BE and Neonates:

The use of base excess (not standard base excess) has also been studied extensively in neonates, by estimating a hemoglobin concentration of 50 g per liter, the estimated hemoglobin concentration in the extracellular fluid, one third of the blood hemoglobin concentration.

SBE is valid for adults only: 5 L of blood diluted in 15 L of extracellular space.

In neonates, however, the extracellular space makes up 40% of their body weight, not 20% as in adults. Therefore, the standard base excess must not be applied to neonates.

At a meeting in Germany in 2004 [4], the following experimental results were discussed:

Simulation of two neonatal blood samples (umbilical artery) by equilibration plus titration and measurement of acid–base status (Roche Diagnostics OMNI 9): pCO_2_ 55.3 and 54.0 instead of 55 mmHg predetermined by equilibration, pO_2_ 15.8 and 16.4 instead of 15 mmHg, BE 15.2 instead of 15.0 and 21.0 instead of 20 mmol/L, when the correct BE calculation formula including sO_2_ (12.4 and 10.8%, respectively) was used.

In contrast, the results for use of the Radiometer formula for the BE of extracellular fluid are -10.1 instead of 15 mmol/L, and −15.3 instead of 20 mmol/L [4].

This shows that **standard base excess (SBE)** is valid for use in only one specific clinical situation, i.e., an adult patient with normal Hb content (g/dL), O_2_ saturation (%), and extracellular space. It cannot be applied to neonates, because their extracellular space is relatively much larger than that of adults, nor to adult patients with hypoxia, anemia, or hypovolemia.

## Base excess—clinical practice—diagnostic tool

### BE and lactate

During shock, anaerobic metabolism is reflected by serum lactate levels. In contrast, the BE is a calculated value that is influenced not only by lactic acidosis. The facts:

1. The change in correctly calculated BE (Δ mmol/L blood), measured in cubital venous blood, is directly proportional to the lactate change (Δ lactate mmol/L plasma, not to the Δ lactate mmol/l blood), demonstrated in 17 athletes during exercise load and recovery [12]. Naturally, the anion lactate is distributed in the whole extracellular space, proportionally to plasma lactate, while the corresponding H^+^ of the lactic acid is buffered mostly by hemoglobin, characterized by the base excess of blood.
2. The same optimal proportionality of lactate vs. base excess was demonstrated in the neonatal clinical situation: the measurement of lactate in the umbilical vein (blood with high sO_2_ from the mother to the fetus) as well as in the umbilical artery (blood with low sO_2_ from the fetus to the mother) resulted in an optimal proportionality of lactate vs. base excess in 278 healthy newborns. This is predicated on the correct calculation of the BE including the sO_2_ [13].
3. The relationship between BE and lactate values can be appreciated from an understanding of how physiological mechanisms within the lungs, liver, and kidneys interact with maintaining the acid–base balance [3]. The contribution of the liver is notable (although many authors regrettably overlook it, e.g., References 2, 3) since it is responsible for the elimination of all acids (H^**+**^ and lactate) and for the continuous production of glucose needed for metabolic activity in the brain.
4. Clinically, when the change in BE is less than proportional to that in cLac, this could be because of a therapy with HCO_**3**_^**−**^ or a solution for infusion containing lactate [14].

Noteworthy is a recent study [15] which compared the capability of alactic base excess (aBE; representing the influence of unmeasured anions) to a combination of BE and lactate, all measured upon admission to the ICU, to predict 28-day mortality of shock patients.

### BE and temperature

Unfortunately, the patient’s temperature has not been mentioned. In vitro studies have demonstrated that BE is independent of temperature [16].

In hypothermic patients, it is recommended to look primarily at the ventilated patient’s BE because the metabolism is diagnosed via a temperature-independent BE [16].

Thus, it is recommended that hypothermic patients be ventilated according to a target value of paCO_2_ 40 ± 5 mmHg under capnometry (end-tidal) monitoring (petCO_2_, mmHg), and that the values obtained in a blood gas analyzer at 37°C be corrected for the patient’s real body temperature using an internally validated algorithm (so-called pH–stat procedure).

### BE and mortality

BE has gained attention as a potential index to assess the risk of mortality in patients with multiple injuries. This is summarized in a review of the literature (Fig. 7, Ref. 18).

Of course, this data cannot establish that base excess—at hospital admission—is indeed the cause of the observed mortality.

**Fig. 1:**
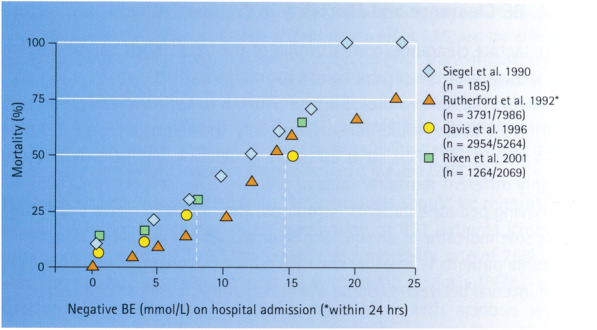
[taken from18] Mortality vs. base excess (BE) in multiple trauma patients: Correlation between mortality (%) and base excess (mmol/L) on hospital admission and 24 h thereafter * in a population of approximately 8200 patients selected from about 15,300 patients [80, 321,326, 347]

**Fig. 2:**
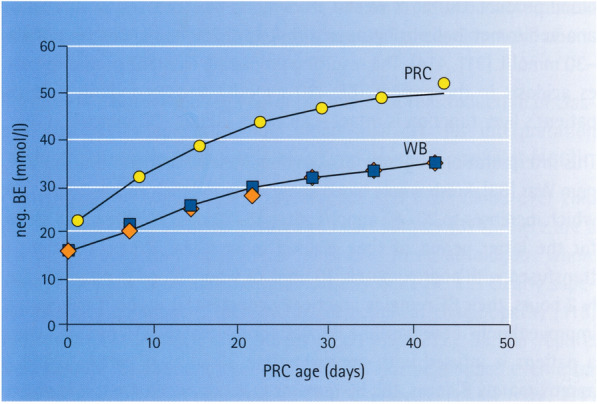
[taken from18] Negative base excess (mmol/L during storage of packed red cell (PRC) or whole blood (WB) units with and without leukocyte depletion [20]

Back to the chapter BE and Lactate:

Within the cited recent study [15] which compared the capability of alactic base excess (aBE) upon admission to the ICU to a combination of BE *plus* lactate, it is important to emphasize the following:

1. A number of 143 shock patients are not comparable with 8,200 multiple trauma patients in Fig. 1, because shock is a special case for the formation of lactates due to a lack of oxygen, i.e., the diagnostic use of lactate as a marker of hypoxia is probably unique.
2. In addition to the time point of ICU admission, the so-called lactate clearance, i.e., the decreasing lactate concentration over hours after the shock event, is an ideal method for assessing the course of the shock.
3. Many clinicians apparently are not aware that the use of lactate-containing infusion fluids (such as Ringer´s Lactate RL with 27 mmol/l lactate or older infusion solutions with up to 45 mmol/l lactate) or blood products (such as packed red cells (PRC) to about 50 mmol/l increasing during storage, as shown in Fig. 2).

### BE and coagulation

In the acute setting, base excess (BE) may also be correlated with the risk of potentially fatal coagulation disturbances in patients with trauma. Coagulopathy in exsanguinating patients is almost always caused by acidosis and hypothermia; this is well documented.

A significant (p < 0.001) correlation between prothrombin level (%) and negative base excess was found—in vivo—in 4066 out of a total of 20,815 severely injured (ISS ≥ 16) multiple-trauma patients in the Trauma Registry of the German Society of Trauma Surgery (Deutsche Gesellschaft für Unfallchirurgie) receiving primary care [14].

These bench and bedside findings therefore suggest that a base deficit of approx. 15 mmol/L reduces clotting activity to approx. 50%, which secondly explains the reported mortality rate of approximately 50% in multiple-trauma patients.

Further studies—in vitro—using three selected coagulation factors have shown that clotting factor activity is primarily determined by pH or BE; clotting factor activity halved at BE −12.5 mmol/L (pH 7.20) and doubled at BE + 16.5 mmol/L (pH 7.60). This observation was recently corroborated in patients in vivo, see above.

To summarize, the pattern of volume or blood component therapy requires an urgent revision [17]: initially, balanced colloids; followed by plasma (volume, coagulation factors, acidosis prevention), and then fresh RBCs.

So far, this article has described use of the base excess (BE) as a calculated value for ***diagnostic use*** related to, e.g., shock, metabolism, temperature, clotting. However, there is another side, too: BE is a useful parameter for various therapies, e.g., administration of sodium bicarbonate or intravenous fluids, i.e., BE for ***therapeutic use***.

## Base excess—therapeutic tool—potential base excess (BEpot)

### BE and solutions for infusion

For this purpose, the diagnostic BE has been modified to obtain the therapeutic BE as follows: BE, defined in analogy to blood, indicates the amount of HCO_3_ required to bring the pH of the (acidic) solution for infusion to the normal pH of 7.4. The BEpot (mmol/L) indicates the amount of HCO_3_ that can potentially be released in the body after infusion and metabolism of anions [18]. This value is calculated by adding BE (with a negative sign) in mmol/L to the sum of metabolizable anions, taking into account their valence.

The concept of potential Base Excess (BEpot; mmol/L) was introduced in 2006 as an index describing the effect of an intravenous fluid on acid–base equilibrium, i.e., whether it has an alkalizing or acidifying effect [18]. Unfortunately, this index has not yet been adopted by manufacturers, which recently prompted an urgent call for them to do so by an interdisciplinary international group of authors [19].

The BEpot, first recommended for solutions for infusion in 2006 [18], is actually accepted for labelling crystalloid and colloid solutions by pharmaceutical companies in Germany.

After infusion and anion metabolism, a solution with a BEpot of 0 mmol/L (e.g., a solution for infusion with 24 mmol/L of acetate instead of HCO_3_) has no effect on the patient’s acid–base balance: neither acidosis nor alkalosis.

In 2022, eleven authors from different countries discussed this BEpot (mmol/l) and came to the following conclusion [19]: “We recommend strongly that the medical community take Lönnqvist’s appeal (’time for a solution’) seriously and urge medical companies and manufacturers to provide improved infusion solutions that are physiologically composed and balanced (Table 1), and which include clear and detailed guidance for their safe and effective use. We believe that these relatively simple steps, which can be achieved without increasing costs, will have a substantial clinical benefit in reducing morbidity and potentially saving lives.”

### BE and blood products

Base excess was found to be superior for detecting hypovolemic shock and stratifying patients in hemorrhagic shock with respect to the need for early transfusion of blood.

The transfusion of erythrocytes in the form of packed red cells (PRCs) is increasingly being viewed critically. A condensed summary of this view is reflected in the title of a 2008 editorial: “New blood, old blood, or no blood?” [6].

Blood products represent the classical field of application for BEpot [18]. Packed red blood cells (PRCs) are known to have a negative BE even at the time of preparation. Theoretically, this BE value would be approximately 20 mmol/L, since bicarbonate normally present in blood (20 mmol/L blood) is almost completely eliminated during the packing process. During storage, usually at 4 °C for a maximum of 42 days [2], the negative BE of the PRCs increases to -50 mmol/L due to the production of lactic acid by anaerobic metabolism (cf. Fig. 14 in Reference 18). Stored whole blood has a baseline BE of approximately –15 mmol/L on account of the alkalizing effect of plasma citrate (20 mmol/L; with a metabolic activity of 60 mmol/L), thus resulting in a BEpot of + 45 mmol/L. In the patient, the infused whole blood causes primary acidosis (BE -30 mmol/L) as well as—if the patient’s liver function is intact—secondary alkalosis (BEpot + 30 mmol/L) (cf. Fig. 14 in Reference 18).

The transfusion of plasma alters the situation. The balance between the acidifying BE of red cells (production process and formation of lactic acid) and the potentially alkalizing effect of citrate within the plasma produces the following result: PRC is an acidifying and plasma an alkalizing product, as practically no alkalizing component of citrate remains in the PRC unit, i.e., 3 mmol/L (14).

## Conclusion

The optimal requirement for clinically practicable diagnostics is: Kiss, i.e., Keep It Simple and Safe. The Base excess BE (mmol/L) is such a *diagnostic tool *in vivo. In everyday clinical practice, a few microlitres of of blood, no matter whether arterial, mixed venous or venous, are sufficient in any blood gass analyzer for optimal diagnostics of each metabolic acidosis or alkalosis. The BE or BD (base deficit) in about 8,000 patients with multiple injuries have demonstrated that BE on ICU admission is indeed the best prognostic indicator for mortality, complication rate, transfusions needs, etc. Additional, the potential base excess (BEpot, mmol/L) as an *therapeutic tool *in vitro for any infusion solution or blood product indicates the amount of bicarbonate that can potentially be released in the body after infusion or transfusion related to the metabolism of anions. Therefore, this outstanding value Base Excess (BE) was named a parameter with exceptional clinical significance.

## Data Availability

Not applicable.

## References

[1] Berend K (2018). Diagnostic use of base excess in acid-base disorders. N Engl J Med.

[2] Langer T, Brusatori S, Gattinoni L (2022). Understanding base excess (BE): merits and pitfalls. Intensive Care Med.

[3] Mertzlufft F, Zander R (1995). The liver: The forgotten organ in acid-base balance. Anästhesiol Intensivmed Notfallmed Schmerzther.

[4] Qualitest No. 8—2005. https://www.physioklin.de/fileadmin/user_upload/physioVARIA/qualitest/quali_h8_2005.pdf

[5] Kofstad J (2001). Base excess: a historical review - has the calculation of base excess been more standardised the last 20 years?. Clin Chim Acta.

[6] Adamson JW (2008). New blood, old blood, or no blood?. N Engl J Med.

[7] Gattinoni L, Pesenti A, Matthay M (2018). Understanding blood gas analysis. Intensive Care Med.

[8] Zander R, Lang W (2004). Letter to the editor: base excess and strong ion difference: clinical limitations related to inaccuracy. Anesthesiology.

[9] Ziegenfuß T, Zander R (2019). Letter: understanding blood gas analysis. Intensive Care Med.

[10] Nöldge-Schomburg G, Armbruster K, Geiger K, Zander R (1995). Experimental studies of acid-base balance and lactate metabolism by the liver. Anästhesiol Intensivmed Notfallmed Schmerzther.

[11] Lang W, Zander R (2002). The accuracy of calculated base excess in blood. Clin Chem Lab Med.

[12] Zander R, Lachtermann E (1999). Optimale Proportionalität zwischen Base Excess des Blutes und Laktatkonzentration des Plasmas. Anästhesiol Intensivmed.

[13] The correct calculation of base excess by the point of care machine (Die korrekte Berechnung des Base Excess im POC-Gerät). https://www.physioklin.de/physiopoc/saeure-basen-sauerstoff-elektrolyt-status/die-korrekte-berechnung-des-base-excess-im-poc-geraet.html

[14] Zander R. Fluid management—second expanded (corrected) edition 2020. Paperback, ISBN: 978–3–347–12851–4. 2023—pdf: www.shaker.de/shop/978-3-8440-8984-4

[15] Smuszkiewicz P, Natalia-Jawien N, Szrama J, Lubarska M, Kusza K, Guzik P. Admission Lactate concentration, base excess, and alactic base. excess predict the 28-day inward mortality in shock patients. J Clin Med 2022; 11: 6125 10.3390/jcm11206125 PMC9604570 36294445

[16] Zander R (2011). Adequate ventilation in hypothermic patients. Appl Cardiopul Pathophysiol.

[17] Zander R (2010). Anaemia and massive bleeding apart from the aspect of oxygenation. Wien Klin Wochenschr.

[18] Zander R (2006). Infusion fluids: why should they be balanced solutions?. EJHP Pract.

[19] Mertzlufft F, Brettner F, Crystal GJ, Hollmann MW, Kasatkin A, Lönnquist PA, Singer D, Sümpelmann R, Wenzel V, Zander R, Ziegenfuß T (2022). Intravenous fluids: issues warranting concern. Eur J Anasthesiol.

[20] Zacharowski K, Zander R (2009). In response to damage control resuscitation 2009. BMJ.

[21] Schaffartzik W (2007). Base excess. Anaesthesist.

